# Poly (Butyl Acrylate)-Graft-Polystyrene Synthesis by Free-Radical Polymerization: Interplay between Structure, Morphology, Mechanical, and Optical Properties

**DOI:** 10.3390/polym11081317

**Published:** 2019-08-07

**Authors:** J. Fage, K. Knoll, N. Niessner, O. Carstensen, T. Schulz, F. Malz, M. Döring, F. Schönberger

**Affiliations:** 1 Fraunhofer Institute for Structural Durability and System Reliability, Plastics Division, Schlossgartenstrasse 6, 64289 Darmstadt, Germany; 2 BASF SE, Carl-Bosch-Straße 38, 67063 Ludwigshafen am Rhein, Germany; 3 INEOS Styrolution Group GmbH, Mainzer Landstrasse 50, 60325 Frankfurt am Main, Germany; 4 LANXESS Deutschland GmbH, Chempark Leverkusen, Gebäude R14, 51369 Leverkusen, Germany

**Keywords:** graft copolymers, free-radical polymerization, polystyrene, poly (butyl acrylate), morphology, toughness, transparency

## Abstract

We report a new method of preparation of poly (butyl acrylate)-g-polystyrene/polystyrene blends by free-radical polymerization. Copolymerization of glycidyl (meth)acrylate with butyl acrylate is followed by a polymer analogous reaction of this copolymer with acrylic acid and subsequent copolymerization of the modified backbone with styrene. Investigation on the number of reactive groups per backbone chain and its molecular weight allows grafting efficiencies of about 35% to be reached, as well as low cross-linking. Blends of nanophase-separated copolymers having a backbone with M_n_ of around 50 kg/mol and 4 reactive groups per chain are transparent, with haze as low as 14%, tensile strength of around 22 MPa, and elongations at the break of around 3%. Correlation between morphology determined by transmission electron microscopy and properties of the blend is established.

## 1. Introduction

Copolymer molecules are known to self-assemble into a variety of ordered structures in the melt and solid state by a process called micro- or nanophase separation. These micro- or nanophase-separated structures endow these copolymers with outstanding mechanical (stiffness, strength, toughness, etc.) and optical (transparency) properties [1]. Copolymers also play a crucial role as compatibilizers or impact modifiers in polymer blends [2].

Combining the properties of antagonist polymers, the new material acquires new properties and applications, as for example, styrene-butadiene-styrene copolymers. These types of copolymers can be used for food packaging or medical devices due to their high flexibility, impact strength, and transparency [3]. Use in biomedical application has also been reported recently [4,5,6].

Poly(butyl acrylate) (PBA) is a polymer with low glass transition temperature and high UV resistance [7]. Polystyrene (PS) is a glassy, amorphous polymer with outstanding clarity, gloss, and processability. However, it suffers from inherent brittleness, limiting its use in application where deformation resistance is required. Therefore, the combination of properties of these two polymers is of great interest.

When styrene is copolymerized with a soft phase forming monomer, such as butyl acrylate or butadiene, immiscibility between the hard and soft parts of the block copolymers causes their segregation into nano-structured domains. The resulting morphology determines their mechanical and optical properties and depends on multiple structural parameters, such as the PS content, the molar mass of the individual blocks, and the architecture of the copolymer [8,9,10].

In order to enhance the toughness of PS, several approaches are commonly used, ranging from rubber-modified polystyrene to a broad range of styrenic copolymers [3]. Incorporation of rubbery particles into the PS matrix is the easiest method to increase toughness. Because of high incompatibility between the phases, the PS-grafted rubbery particles are dispersed in the PS matrix. Elastomer particles initiate the deformation process, stabilize crack propagation, and decrease the detrimental dilatational stress field in the matrix by internal voiding [4]. In such blends, toughness is influenced by the compatibility between PS and the elastomer phase, and by the particle size of the dispersed phase. High-impact polystyrene (HIPS) is made by radical polymerization of styrene containing dissolved polybutadiene (PB). The polymerization induces a phase separation and a phase inversion, which results in the well-known salami morphology of HIPS, with its superior impact strength compared to homo-PS [9]. However, the typical size of the salami domains of about 2 μm results in opaque products.

In order to combine transparency and impact resistance, the refractive indices of both phases must be matched or the domain sizes must be well below the wavelength of the visible light [5]. 

Several synthesis approaches to tailor copolymer structures are known in the literature, such as atom transfer radical polymerization (ATRP), reversible addition fragmentation chain transfer (RAFT), nitroxide mediated polymerization (NMP), or anionic polymerization methods [8,10,11,12,13]. ATRP and RAFT are efficient methods for designing multi-block copolymers, such as poly (butylacrylate)-g-polystrene (PBA-g-PS) or poly (butylacrylate)-g-poly(methyl methacrylate) (PBA-g-PMMA) [11,12,14,15,16,17,18,19]. Block copolymers and polymers with more complex architectures have been developed by anionic polymerization [20,21]. It was found that the architecture of such copolymers has a strong impact on their nano-structure, and hence the mechanical and optical properties. Graft copolymers differ in their morphology formation in comparison to block copolymers. In principle, graft copolymers with high PS content form a lamellar morphology, while block copolymers with the same PS content prefer cylindrical ones. Adhikari et al. studied the behavior of star-shaped PS copolymers in comparison to the common styrene-butadiene triblock copolymer structure [22]. At similar ratios of soft and hard phases, star-shaped copolymers form a co-continuous morphology, while triblock copolymers have a lamellar one. Consequently, star-shaped copolymers with volume fractions of only 25% soft phase show tensile strength and elongation at the break similar to triblock copolymers containing 50% soft phase.

Controlled polymerization methods, although proven to be powerful in the design of well-defined nanostructures, have some major drawbacks. Anionic polymerization requires high purity of solvents, monomers, and atmosphere, and is limited, especially when acrylate based monomers are used [23,24,25]. The use of ATRP or RAFT polymerization is advantageous in terms of monomer choice and purification needed compared to anionic polymerization. However, conversion of monomers is usually low and high molecular weight is difficult to obtain. 

In contrast, free-radical polymerization offers numerous advantages over the controlled polymerization methods. It allows use of a large variety of monomers and does not require demanding conditions in terms of reagent and environment purity. The process is simple, cost-effective, and attractive for industrial use. Free-radically induced grafting of styrene onto anionically polymerized PB to yield tough and transparent copolymers with nanolamellar morphology is, for example, reported by Portl [26]. Using PBA as a backbone instead of PB is expected to increase the blend UV resistance. 

The aims of our current work are (i) to develop a synthetic approach for PBA-g-PS by using free-radical polymerization for both the backbone and the side chains, (ii) to investigate the influence of selected structural parameters on the copolymerization process, and (iii) to correlate their mechanical and optical properties with their morphology.

## 2. Materials and Methods 

### 2.1. Materials

Butyl acrylate (BA) and styrene were destabilized by being passed through basic aluminum oxide to remove traces of inhibitor. They were kept below 5 °C before use. Benzoyl peroxide (BPO) was purified by recrystallization in methanol and kept at −20 °C before use. Acrylic acid (AA), triphenylphosphine (TPP), 4-methoxyphenol (MEHQ), glycidyl methacrylate (GMA), tert-dodecanethiol (TDT), toluene, cyclohexane (HPLC grades), and methanol (technical and HPLC grades) were used without any further purification. All the chemicals were supplied by VWR Chemicals. Glycidyl acrylate (GA) was supplied by ABCR GmbH and used without purification.

#### 2.1.1. Copolymerization of PBA-co-GMA and PBA-co-GA

A 250 mL three-neck round bottom flask was equipped with an overhead stirrer (250 rpm), a condenser, and a nitrogen inlet. Then, 49.04 g of BA and 0.96 g of GMA or GA and 0.38 g of tert-dodecanethiol were dissolved in 50 g of cyclohexane and the mixture was heated up to 83 °C. The amounts of tert-dodecanethiol, BPO, and GMA (or GA) were varied (see Table 1). Then, 0.50 g of benzoyl peroxide was added to the mixture and stirred for 1 h. After cooling down to RT, the polymer was precipitated from the solution in a minimum of 5 folds of a mixture of methanol and water (*v*/*v*: 90/10). The polymer was re-dissolved in THF and the precipitation procedure was repeated. The polymer was dried at 70 °C under vacuum until constant weight was reached.

#### 2.1.2. Post-Modification of PBA-co-GMA and PBA-co-GA

A 250 mL three-neck round bottom flask equipped with an overhead stirrer (250 rpm), a condenser, and a nitrogen inlet was charged with a solution of 50 g of PBA-co-GMA in 50 g of toluene. Subsequently, a solution containing 10 g of acrylic acid, 1 g of triphenylphosphine, and 0.1 g of 4-methoxyphenol was added to the flask. The mixture was then heated up to 110 °C for 64 h. The polymer was precipitated in a minimum of 5 folds of a mixture of methanol and water (*v*/*v*: 90/10). Afterwards, the polymer was re-dissolved in THF and precipitated twice using the same procedure. The polymer was then dried at 50 °C under vacuum until constant weight was reached.

#### 2.1.3. Graft Copolymerization

A 500 mL reactor flask equipped with an overhead stirrer (500 rpm), a condenser, and a nitrogen inlet was filled with 18 g of post-modified backbone, 72 g of cyclohexane, and 72 g of styrene. The solution was heated to 80 °C and 0.12 g of BPO was added to the mixture. Subsequently, a solution of 0.48 g of BPO in 4.31 g of styrene was dosed for 6 h. After 2 h, 72 g of styrene was slowly added to the reaction mixture within 4 h. After 6 h of reaction, the polymer was isolated by precipitation in a minimum of 5 folds of methanol. The polymer was re-dissolved in THF and precipitated in a minimum of 5 folds of methanol. The polymer was then dried at 60 °C under vacuum until constant weight was reached.

#### 2.1.4. Chemical Characterization of Copolymers Blends

**Size exclusion chromatography**

Size exclusion chromatography (SEC) was performed on an Agilent Series 1100 instrument (Agilent Technologies, Inc., Santa Clara, California, United States) equipped with a HPLC pump, a series of columns of PL gel mixed C (1× guard column and 2 × 300 × 7.5 mm) (Agilent), a refractive detector, and a UV detector. THF was used as solvent at 35 °C with a flow rate of 1 mL/min and the injection volume was 100 μL. Calibration was performed with a series of PS standard from PSS polymer (5 samples between 0.580 and 2536 kDa)

Deconvolution of the SEC curves was performed as already reported by Huang et al. [27] using the software Peakfit 4.12 (Systat Software, Inc., San Jose, California, United States). The UV elugram of the blend was separated into three peaks, with two corresponding to the copolymer in low elution volume and one corresponding to the homo-PS in high elution volume.

The grafting efficiency (GE) was defined as the mass of the grafted PS divided by the total PS produced. As the mass of PS is linked to the area under the SEC curves after fitting, the grafting efficiency can be calculated as:

(1) $$GE=\frac{I_{co}}{I_{co}+I_{homo}}$$

where I_co_ is the integral of the copolymer peak and I_homo_ is the integral of the homo-PS peak. Each integral was determined from peak fitting after deconvolution. 

**NMR spectroscopy**

The ^1^H NMR spectra were recorded at room temperature in deuterated chloroform with a Bruker 300 spectrometer operating (Bruker Corporation, Billerica, Massachusetts, United States)) at 300 MHz. Typical parameters for the proton spectra are a 13.4 μs pulse width, a pulse delay of 5 s, an acquisition time of 2.7 s, a 6000 Hz spectral width, and 128 scans.

The mole fraction of comonomer in the copolymer (x_coM_ in mol.%), the mass fraction of GA or GMA in the copolymer (w_coM_ in wt %), and the number of comonomers per chain (N_coM_) were calculated using the following formulas:

(2) $$x_{\mathrm{coM}}\;=\frac{\frac{I_{\mathrm{coM}}}{N_{H,\;\mathrm{coM}}}}{\frac{I_{\mathrm{coM}}}{N_{H,\;\mathrm{coM}}}+\frac{I_{\mathrm{BA}}}{N_{H,\;\mathrm{BA}}}}$$

(3) $$w_{\mathrm{coM}}=\frac{x_{\mathrm{coM}}\times M_{\mathrm{coM}}}{\left(1-x_{\mathrm{coM}}\right)\times M_{\mathrm{BA}}+x_{coM}\times M_{\mathrm{coM}}}$$

(4) $$N_{\mathrm{coM}}\;=\frac{w_{\mathrm{coM}}\times M_{n\left(\mathrm{backbone}\right)}}{M_{\mathrm{coM}}}$$

where I_coM_ and I_BA_ represent the NMR integration area of the GA (2.59 ppm) or GMA (2.63 ppm) peak and BA peak (2.25 ppm), respectively. N_H,coM_ and N_H,BA_ represent the amount of hydrogen corresponding to the integrated peaks in GA or GMA and BA, respectively. M_coM_ and M_BA_ represent the molecular mass of GA (128.13 g/mol) or GMA (142.15 g/mol) and BA (128.17 g/mol) repeating units. M_n(backbone)_ is the average molecular weight of the backbone determined by SEC analysis.

In a similar manner, the mole and mass fraction of acryloyl comonomer after the post-modification reaction of the backbone, as well as the number of acryloyl comonomer per chain, were calculated as follows:

(5) $$x_{\mathrm{Acr}}\;=\frac{\frac{I_{\mathrm{Acr}}}{N_{H,\;\mathrm{Acr}}}}{\frac{I_{\mathrm{Acr}}}{N_{H,\;\mathrm{Acr}}}+\frac{I_{\mathrm{BA}}}{N_{H,\;\mathrm{BA}}}}$$

(6) $$w_{\mathrm{Acr}}=\frac{x_{\mathrm{Acr}}\times M_{\mathrm{Acr}}}{\left(1-x_{\mathrm{Acr}}\right)\times M_{\mathrm{BA}}+x_{\mathrm{Acr}}\times M_{\mathrm{Acr}}}$$

(7) $$N_{\mathrm{Acr}}\;=\frac{w_{\mathrm{Acr}}\times M_{n\left(\mathrm{Backbone}\right)}}{M_{\mathrm{Acr}}}$$

where I_Acr_ represents the integration area of the acryloyl comonomer peak at 6.41 ppm, M_Acr_ and M_BA_ represent the molecular mass of the acryloyl monomer (199.17 g/mol when using GA monomer and 214.21 g/mol when using GMA and BA repeating unit (128.17 g/mol). From this value, the theoretical number of monomers between two reactive groups N_m_ is calculated:

(8) $$N_{m}=\frac{\frac{M_{n\left(\mathrm{Backbone}\right)}}{M_{\mathrm{BA}}}}{N_{\mathrm{Acr}}}$$

**Non-soluble fraction**

The non-soluble fraction (NS (%)) of the reaction products was determined from Soxhlet extraction in toluene for 64 h. The extraction thimble was weighted empty (w_0_) and with the polymer powder (w_1_). After extraction, the thimble was dried for 48 h at 60 °C under vacuum and weighted again (w_2_). The non-soluble fraction was calculated from the following equation:

(9) $$\mathrm{NS}\;\left[\text{\%}\right]=\;\frac{w_{2}-w_{0}}{w_{1}-w_{0}}\;\times 100$$

#### 2.1.5. Film Casting Characterization

**Film casting**

Films were prepared in 10-cm diameter petri dishes from 20 mL of a 3 wt.% polymer solution in toluene. Toluene was evaporated by keeping the petri dish in a fume hood for 24 h at RT. The resulting films were removed from the petri dish by dipping them into deionized water for 2 h. Prior to characterization, the films were dried at 50 °C under vacuum for 24 h. Film thickness was measured with a micrometer caliper. All of the films had a mean thickness of 100 ± 10 m. Dumbbell-shape specimens were punched out from the film for mechanical testing.

**Mechanical characterization**

Stress-strain curves were measured using a Zwick Roell Z 2.5 tensile tester (ZwickRoell GmbH & Co.KG, Ulm, Germany) at 23 °C and 48% relative humidity. The samples were conditioned for 2 days prior to testing. The test was performed at a rate of 0.1 mm/min below 0.25% of elongation to determine Young’s modulus (E). The rest of the test was performed at a rate of 20 mm/min to determine tensile strength (TS) and elongation at the break.

**Optical characterization**

Optical properties including haze, transmittance, and clarity were measured on solvent-cast films using the Haze Gard plus (BYK-Gardner GmbH, Geretsried, Germany).

**Morphological characterization by TEM**

The morphology of the samples was investigated by transmission electronic microscope (TEM, 60 kV, Zeiss EM10, Carl Zeiss AG, Oberkochen, Germany). Ultrathin sections of the samples (approximately 40 nm) were microtomed from a specimen embedded in epoxy resin using a UTC ultramicrotomy device (Leica Microsystems GmbH, Wetzlar, Germany). The temperature of the chamber and of the blade was fixed at −20 °C and the temperature of the sample was −50 °C.

## 3. Results and Discussion 

### 3.1. Synthetic Approach

**Scheme 1:**
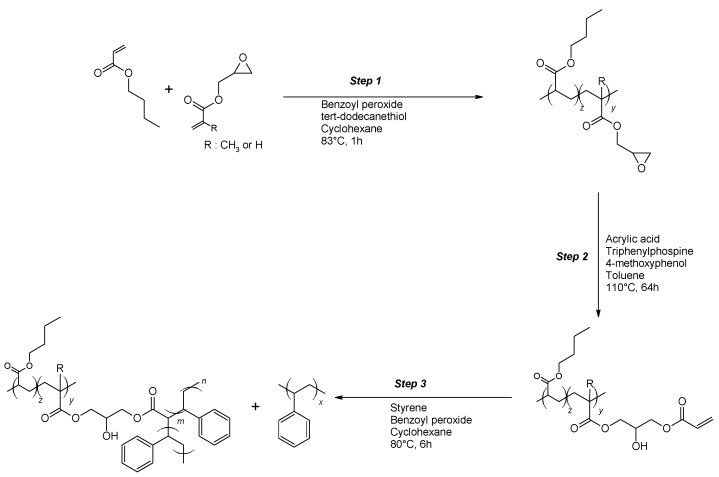
Synthesis of PBA-g-PS and PS.

Unlike polybutadiene (PB) that Portl used to synthesize their PB-g-PS/PS blends resulting in lamellar nano-morphology, polybutylacrylate (PBA) does not have any reactive sites for (controlled) grafting [26]. Therefore, appropriate backbones for grafting with styrene were synthesized in the first step. According to Scheme 1, this backbone was made in a two-step process consisting of (i) copolymerization of butyl acrylate (BA) with glycidyl acrylate (GA) or glycidyl methacrylate (GMA) and (ii) post-modification of the PBA-co-GA or PBA-co-GMA copolymers through reaction with acrylic acid. Styrene was then copolymerized in the presence of the reactive backbone, leading to blends of PBA-g-PS and PS. 

In the next section, the influence of structural variations in the backbone (molecular weight, type, and amount of comonomer) on the copolymerization with styrene is investigated. 

The following nomenclature is used for the (modified) backbones and the graft copolymers. Backbones are named as BXX(M_n_-wt.%), with XX indicating the type of comonomer used (GA or GMA), M_n_ is the average molecular weight (in kg/mol, determined from SEC), and wt.% is the weight percentage of the comonomer in the backbone (determined from NMR). The post-modified backbones are named as BXX(M_n_-N_Acr_), with XX indicating the type of comonomer used, M_n_ is the average molecular weight (in kg/mol, determined from SEC), and the number of acryloyl groups per chain of the modified backbone N_Acr_. The graft copolymers are indicated as G-BXX(M_n_-N_acr_). 

### 3.2. Backbone Synthesis: Molecular Weight and Functionality Variation

First, the influence of both the molecular weight of the PBA backbone and the number of reactive sites on the backbone is studied. In order to regulate the molecular weight of the backbone and to minimize uncontrolled cross-linking due to hydrogen abstraction [28], tert-dodecanethiol (TDT) is used as a chain-transfer agent during the copolymerization of BA with GMA or GA.

Acrylate and methacrylate have different copolymerization parameters with BA. GMA copolymerizes with BA at a ratio of 2.15/0.12 [29]. This means that a composition drift appears during copolymerization: GMA-rich chain segments are formed at the beginning of the reaction, while BA-rich chains form toward the end of the reaction. No copolymerization parameters are available for GA and BA in the literature, but it is reported that hydroxy ethyl acrylate (which is structurally similar to GA) copolymerizes with BA at a ratio of 0.94/0.23 [30]. Therefore, it is obvious that GA will also copolymerize with BA with lower composition drift compared to GMA. 

Therefore, structural variations of the backbone in terms of molecular weight and in terms of the density of reactive groups (for grafting) are studied by varying the amount of GMA or GA, tert-dodecanethiol (TDT), and BPO. Table 1 summarizes the effect of these variations on the molecular weight and the number of comonomers per chain (N_coM_). 

The variation of TDT (while keeping BPO constant) in the system allows control of the molecular weight. With 0.75 wt.% TDT relative to the amount of monomers, a molecular weight around 30 kg/mol is obtained (B_GMA_(26-1.9) and B_GMA_(35-9)). Reducing the TDT value to 0.25 wt.% increases the molecular weight to around 50 kg/mol. For a further increase of the molecular weight of the backbone, the amount of BPO is decreased to 0.1 wt.% and the TDT amount is fixed at 0.2 wt.%, resulting in a molecular weight of around 100 kg/mol. 

**Figure 1:**
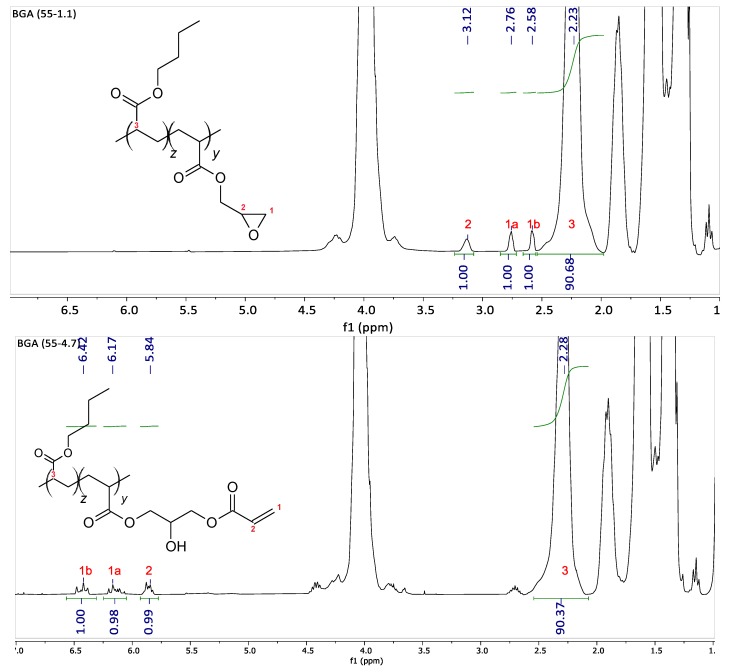
The ^1^H NMR spectra of backbone B_GA_ (55-1.1) before and after modification.

The amount of comonomer measured by NMR corresponds to the feed value, as the polymerization conversion reaches nearly 100%. After post-modification, the number of acryloyl functions is measured by NMR. As depicted in Figure 1 for the backbone B_GA_(55-1.1), the glycidyl functions in the backbone appearing between 2.6 and 3.2 ppm are completely converted to acryloyl functions between 5.8 and 6.4 ppm. All of these polymers are fully soluble in toluene, indicating that no cross-linking occurred in the backbone polymerization step or during its modification.

Table 2 shows the number of acryloyl groups per chain after modification of the backbone and the mean distance between adjacent acryloyl groups.

Table 2 provides evidence for the quantitative formation of post-modified backbones, as the number of acryloyl groups per chain is equal to the number of glycidyl groups. It is worth mentioning that the molecular weight is unchanged before and after the modification of the backbone and that all of the modified backbones are completely soluble in toluene. This means that degradation in molecular weight and cross-linking during post-modification could be avoided. All of the modified backbones are copolymerized with styrene, as described in the next section. 

### 3.3. Graft Copolymerization

One of the aims of the copolymerization of modified-PBA with styrene is a balance between minimum cross-linking of the product and sufficient hard phase content. Dosing of the initiator in the reaction mixture allows a constant concentration in initiator radicals. This principle is applied during the graft copolymerization of styrene in the presence of the modified PBA-co-GMA (or PBA-co-GA) backbones to minimize the risk of cross-linking. In order to reduce the probability of cross-linking further, styrene is added in a large excess during the copolymerization. The ratio between PBA backbone and styrene is fixed to 1 to 8. This allows a conversion sufficient to have around 60 wt.% of PS (grafted or not) in the blend after 6 h of reaction.

#### 3.3.1. Influence of the Distance between Acryloyl Groups 

Table 3 summarizes the non-soluble fraction and molecular weight of the PBA-g-PS made from the various GMA-based backbones. It should be mentioned that the number-average molecular weight is analyzed by SEC, meaning that only the soluble fraction of the sample is captured. As shown in Table 3, the non-soluble fraction of the various samples varies from 0% to 93%. Therefore, M_n_ of the samples G-B_GMA_(35-22) and G-B_GMA_(50-16.7) are not determined, as these samples have high amounts of non-soluble fraction (93 and 73 wt.% respectively), which is not accessible by SEC. 

**Figure 2:**
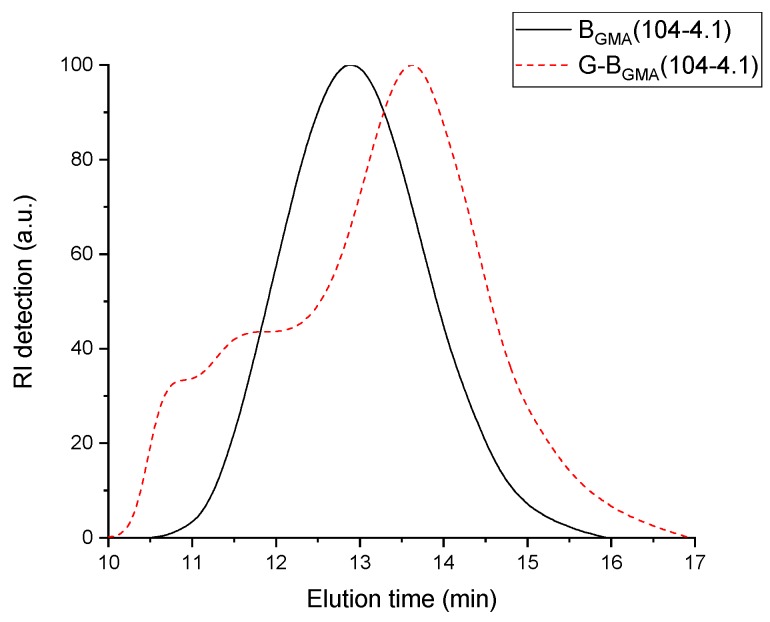
RI elugram of B_GMA_(104-4.1) before and after graft copolymerization.

Comparing G-B_GMA_(26-3.5) with G-B_GMA_(52-4) shows a decrease of the non-soluble fraction from 50 wt.% to 17 wt.% by doubling the number of monomer moieties between adjacent reactive group N_m_. A similar observation is made by comparing G-B_GMA_(50-16.7) with G-B_GMA_(52-4)—the non-soluble fraction is significantly reduced from 73% to 17% when the amount of reactive groups is lowered from 16.7 to 4. At the same time, the backbone length is kept constant at about 50 kg/mol. The backbones with high numbers of reactive sites on short backbones (G-B_GMA_(35-22) and G-B_GMA_(50-16.7)) even lead to highly insoluble products with non-soluble fractions higher than 70 wt.%. This is due to the high reactivity of acryloyl groups. Sample G-B_GMA_(104-4.1) without any cross-linked particles has M_n_ around 52 kg/mol. This value is significantly lower than the molecular weight of its backbone with M_n_ around 104 kg/mol. The corresponding RI signal of the SEC elugram shown in Figure 2 is separated in two areas: one area at low elution volume and another one at high elution volume. Obviously, a fraction of the blend has lower molecular weight than the backbone (higher elution time) and the other fraction has higher molecular weight (lower elution time). The lower molecular weight corresponds to homo-PS formed during the synthesis and the higher molecular weight is attributed to the graft copolymer. This explains why the number-average molecular weight M_n_ of the blend is lower than the molecular weight of the backbone used. Furthermore, the dispersity of the blend is between 4.2 and 5.2. This value of dispersity above 4.0 is obviously due to the broad peak (homo-PS + copolymer), as observed in Figure 2.

As summarized in Table 3, the amount of PS in the blend (grafted or not) varies from 54 to 62 wt.% for the samples with non-soluble fraction below 50%. The amount of PS in the samples with a higher amount of non-soluble fraction (G-B_GMA_(35-22) and G-B_GMA_(50-16.7)) are not measured, as the samples are poorly soluble in NMR solvent. The differences in the amount of PS for the samples are explained by their difference in solubility.

#### 3.3.2. Influence of the Type of Comonomer: GMA vs. GA

**Figure 3:**
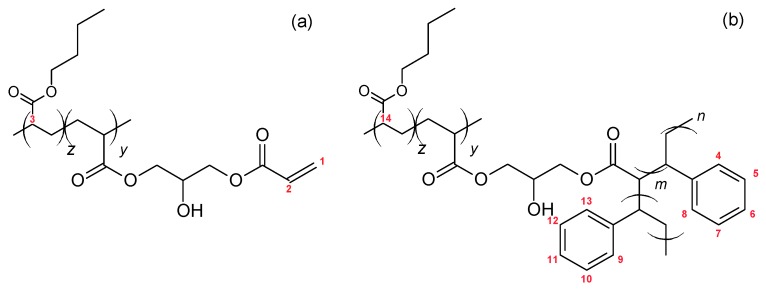
Structure of post-modified backbone (**a**) and graft copolymer (**b**).

Different moieties are present in the blend after graft copolymerization: homo-PS, PBA-g-PS, and possibly unreacted backbone functions. The latter two are shown in Figure 3. 

**Figure 4:**
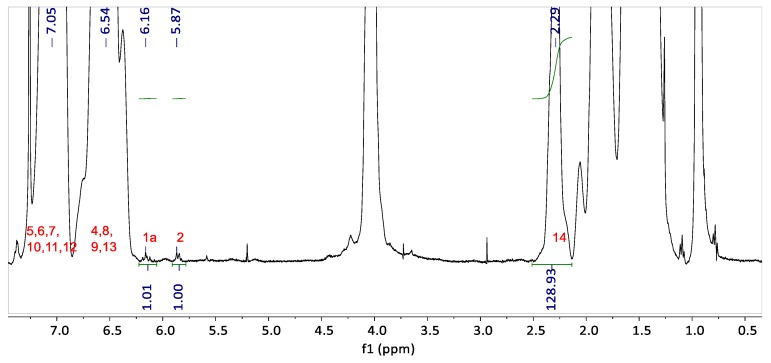
The ^1^H NMR of G-B_GA_(55-4.7).

The ^1^H NMR spectrum of the graft copolymer blend G-B_GA_(55-4.7) is presented in Figure 4. The peaks corresponding to the H nuclei of the PS aromatic ring (H atoms attached to carbon 4 to 13 in Figure 3b) appear between 6.25 and 7.5 ppm. The acryloyl peak from unreacted groups in the backbone (H atoms attached to carbon 1 and 2 in Figure 3a) is detected at 5.87 ppm. 

The integrals of the butyl acrylate H nuclei at 2.29 ppm in comparison to the H nuclei of the acryloyl group at 5.87 ppm are increased compared to the pure backbone (in Figure 1). This means that a fraction of acryloyl groups is consumed during the graft copolymerization. The NMR analysis reveals a grafting efficiency of 28.5% for G-B_GA_(55-4.7). 

A comparison between copolymer blends based on backbones varying by their type of comonomer is presented in Table 4.

Although the number of monomers between two reactive groups of N_m_ is slightly higher in case of G-B_GMA_(52-4) with 101 compared to G-B_GA_(55-4.7) with 91, the amount of non-soluble fraction is decreased from 17 wt.% for the GMA-derived graft copolymer to 0 wt.% for the GA-derived graft copolymer. This is explained by the different reactivity of the comonomer used for the copolymerization of the backbone. When GMA is used as the comonomer, GMA-rich chain segments are formed at the beginning of the polymerization, while BA-rich chains are formed at a later stage. After post-modification and during the graft copolymerization procedure, the acryloyl-rich chain segments have higher probability for cross-linking. This effect is reduced when GA is used as the comonomer as the composition drift during the backbone synthesis is lower.

Samples G-B_GMA_(104-4.1) and G-B_GA_(98-4), which have similar N_m_ around 195, both have 0% non-soluble fraction. This means that not only does the change of comonomer used for the synthesis of the backbone play a role in the amount of non-soluble fraction, but also the distance between the two reactive sites.

The molecular weight of the graft copolymers varies between 48 and 54 kg/mol, independent from the molecular weight of the backbone. As previously explained, the molecular weight of the blend around 50 kg/mol is due to a mixture of high molecular weight graft and low molecular weight homo-PS.

The molecular weight of the blend G-B_GMA_(52-4) is 48 kg/mol, and thus slightly lower than that for G-B_GA_(55-4.7) at 54 kg/mol. This is explained by the amount of non-soluble fraction in G-B_GMA_(52-4), which is not accessible by SEC, and that is attributed to cross-linked particles.. 

#### 3.3.3. Evaluation of Grafting Density by SEC Deconvolution

Graft copolymerization by free-radical polymerization leads to the formation of graft copolymers and homo-PS. SEC deconvolution is used to separate the graft copolymers from the homo-PS due to their difference in molecular weight. 

**Figure 5:**
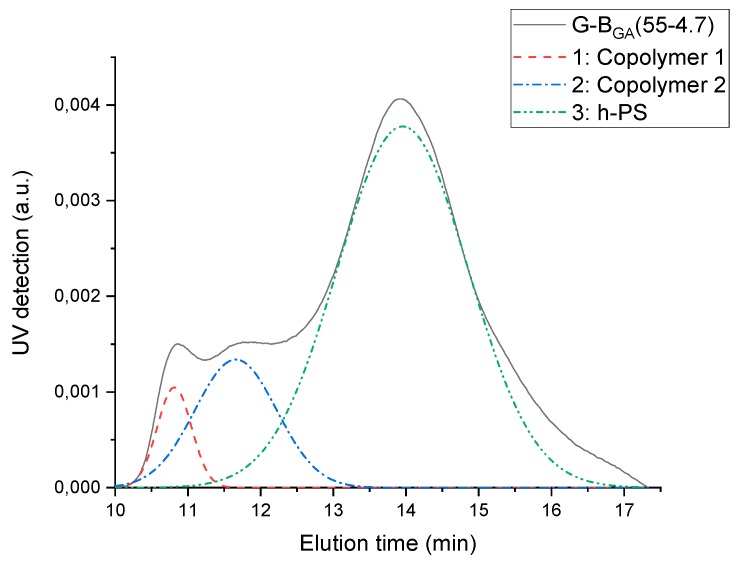
UV elugram of G-B_GA_(55-4.7) and its deconvolution.

The elugram of G-B_GMA_(55-4.7) blend is shown in Figure 5 (black curve). UV detection is used, as polystyrene is UV active while PBA is not. The elugram consists of two zones: one between 11 and 13 min corresponding to high molecular weight graft copolymer, and another one between 13 and 17 min corresponding to homo-PS. The deconvolution method is used to mathematically separate overlapping peaks. It appears from the fitting performed on the sample G-B_GMA_(52-4) that the peak assigned to graft copolymers is further split into two separate peaks, as reported for PB-g-PS/PS blends previously by Huang et al. [27].

**Figure 6:**
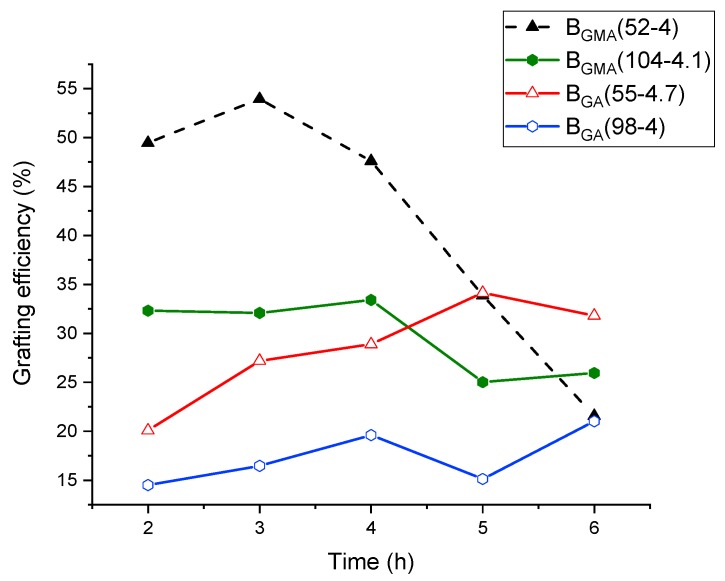
Grafting efficiency of blends through the reaction course.

The course of grafting efficiency of the various GA and GMA-derived backbones with different molecular weights but similar number of acryloyl groups is depicted in Figure 6.

In this series, the sample of blend G-B_GMA_(104-4.1) after 6 h reaction time was the only one that was not fully soluble (non-soluble fraction = 17 wt.%). Therefore, only the grafting efficiency in the soluble fraction is considered for this copolymer blend. All the other samples were completely soluble at all the reaction times so that the grafting efficiency is representative for the entire sample.

The blend G-B_GMA_(52-4) shows a moderately increasing grafting efficiency up to 3 h, followed by a decrease from 3 to 6 h from about 45% after 3 h to 20% after 6 h. The intense decrease from 3 to 6 h is attributed mainly due to the increasing cross-linking fraction of the sample. The grafting efficiency of G-B_GMA_(104-4.1) over reaction time decreases between 3 h and 6 h, although no cross-linking reaction occurred. In comparison, the samples G-B_GA_(55-4.7) and G-B_GA_(98-4) show a slow increase of grafting efficiency through the polymerization. On the other hand, the blends formed with GA-based backbones show a slow increase of the grafting efficiency from the beginning until the end of the reaction. 

This difference is related to the distribution of reactive groups along the backbone chain. As previously mentioned, the reactivity ratio for the copolymerization of BA with GMA induces a composition drift. This means that the backbones based on GMA have a fraction of chains with a high degree of reactive sites, which react from the beginning of the graft copolymerization to form copolymers. Once the reactive sites are consumed, mainly homo-PS is formed.

Moreover, it is observed that the grafting efficiency at 1 h is lower in the case of the GA-based backbone compared to the GMA-based one. However, during the polymerization, the grafting efficiency decreases with the GMA-based backbone and increases with the GA-based backbone and the curves cross or converge after 6 h of reaction. This is the case between B_GMA_(52-4) and B_GA_(55-4.7) and between B_GMA_(104-4.1) and B_GA_(98-4).

By doubling the molecular weight of the backbone while keeping the amount of reactive group constant, the grafting efficiency is also reduced (B_GMA_(52-4) vs. B_GMA_(104-4.1) and B_GA_(55-4.7) vs. B_GA_(98-4)). This is due to the decrease of reactive site density, lowering the probability of grafting. 

#### 3.3.4. Mechanical and Optical Properties

Mechanical and optical properties are determined from solvent cast films of the blends after 6 h of reaction and gathered in Table 5.

Comparison between G-B_GMA_(52-4) and G-B_GMA_(104-4.1) reveals an increase of the backbone molecular weight, keeping the number of reactive groups identical. This effect reduces the transparency of the blends with increase in haze from 23% to 42% and decrease of clarity from 92% to 39%. On the other hand, tensile strength and elongation at break are increased. Comparison between G-B_GMA_(52-4) and G-B_GMA_(104-4.1) also shows a decrease in transparency when the molecular weight of the backbone is increased. Sample G-B_GA_(98-4) is too brittle to run tensile testing. 

When G-B_GMA_(52-4) and G-B_GA_(55-4.7) are compared, it can be seen that the blends prepared with the GA-based backbone has a slightly higher transparency with haze at 14%, compared to 23% with the GMA-based backbone. E modulus and tensile strength are also increased. Elongation of both samples lies in the same range, at 3.4% and 3.0% for types. 

The tendency is different when comparing the blends based on backbones with a higher molecular weight of around 100 kg/mol. Indeed, G-B_GMA_(104-4.1) has higher transparency than G-B_GA_(98-4) with haze at 42%, compared to 70% for the GA-based blend. In terms of mechanical properties, the sample G-B_GA_(98-4) is too brittle for tensile testing, while G-B_GMA_(104-4.1) is elongated at the break at 3.6%. 

Doubling the number of acryloyl groups per chain and keeping the molecular weight constant (G-B_GA_(98-4) and G-B_GA_(98-9.8)) decreases haze from 70% to 60%, but also clarity from 17% to 8%. However, elongation at the break remains quite low at 1.3%, while E modulus and tensile strength are in the same range as for the GMA derived samples.

#### 3.3.5. Morphology

**Figure 7:**
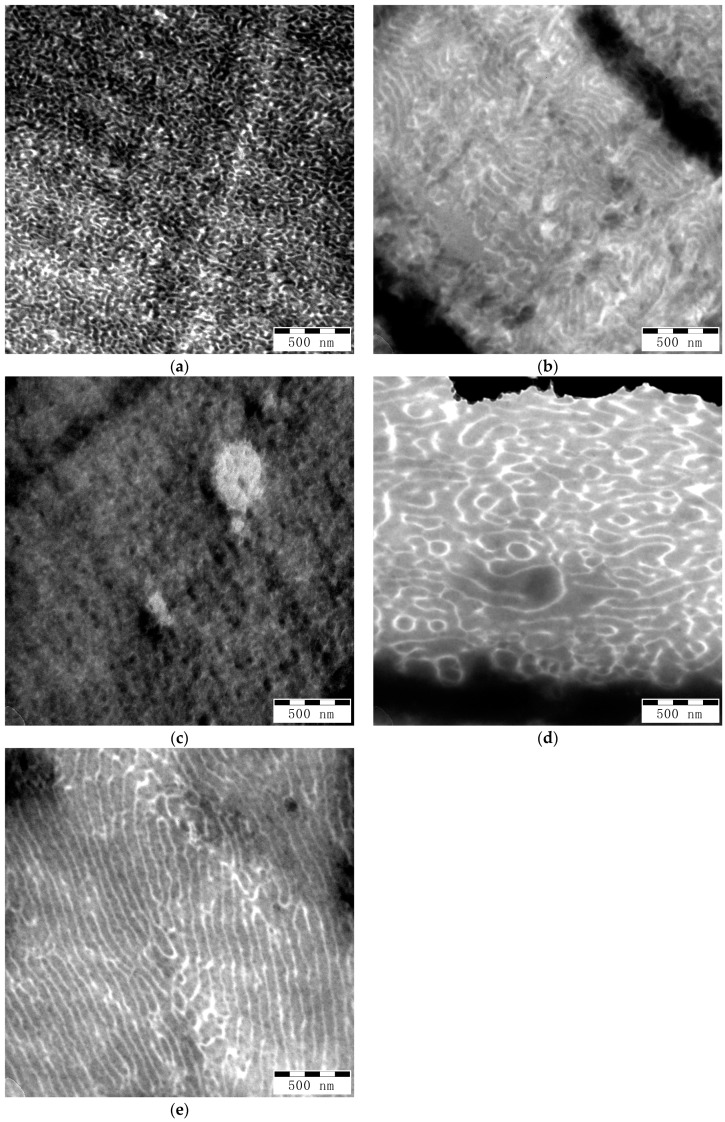
TEM pictures of copolymer blends at magnification of 40,000×. (**a**) G-B_GMA_(52-4); (**b**) G-B_GMA_(104-4.1); (**c**) G-B_GA_(55-4.7); (**d**) G-B_GA_(98-4); (**e**) G-B_GA_(98-9.8).

Mechanical and optical properties of polymer blends strongly depend on their morphology. TEM images presented in Figure 7 show different morphologies in the films made from the various GA and GMA-derived backbones with different molecular weights but a similar number of acryloyl groups. PS phases appear dark, while PBA phases appear bright. Strongly dark areas on the images correspond to breaks or thick areas on the films. Samples G-B_GMA_(104-4.1) and G-B_GA_(98-9.8) in Figure 7b,e show lamellar morphology, while samples G-B_GMA_(52-4), G-B_GA_(55-4.7) and G-B_GA_(98-4) in Figure 7a,c,d rather have co-continuous morphology.

There is an evident difference in the size of domains between the samples. G-B_GMA_(52-4), G-B_GMA_(104-4.1), and G-B_GA_(55-4.7) have PS domains below 50 nm, while G-B_GA_(98-4) and G-B_GA_(98-9.8) have larger PS domains up to about 100 nm. 

The reduced domain sizes in G-B_GMA_(52-4), G-B_GMA_(104-4.1), and G-B_GA_(55-4.7) correlate well with the mechanical characteristics of the films (see previous section). Samples with larger domain size (G-B_GA_(98-4) and G-B_GA_(98-9.8)) have the lowest elongation at break and the samples G-B_GMA_(52-4), G-B_GMA_(104-4.1), and G-B_GA_(55-4.7) have higher ones.

However, none of the PBA-g-PS/PS samples has polystyrene domains below the critical thickness of 20 nm, which are typical values of highly flexible films reported for anionically polymerized butadiene styrene copolymers or the combination of anionic polymerized PB and free-radical grafting of styrene [22,26]. A similar observation is made for copolymers with high flexibility prepared by RAFT polymerization [19].

The difference in optical behavior between G-B_GMA_(52-4)/G-B_GA_(55-4.7) and G-B_GA_(98-4)/G-B_GA_(98-9.8) cannot be traced back to differences in the domain sizes seen in Figure 7 at magnification of 40,000×. All of the shown domain sizes are well below 100 nm, and thus below the wavelength of the visible light. 

**Figure 8:**
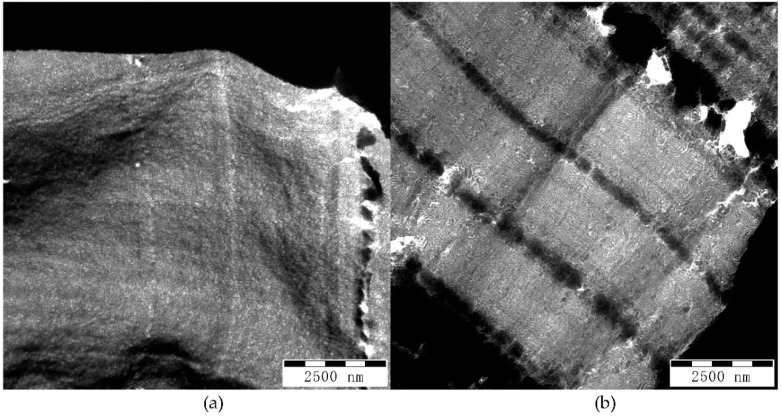

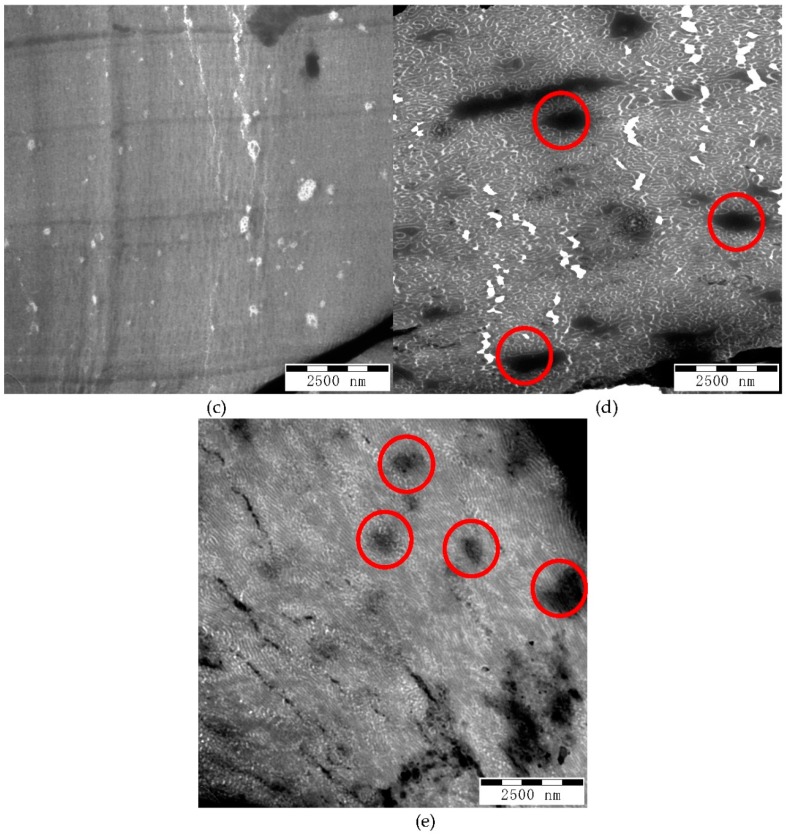
TEM pictures of blends at magnification 10,000×. (**a**) G-B_GMA_(52-4); (**b**) G-B_GMA_(104-4.1); (**c**) G-B_GA_(55-4.7); (**d**) G-B_GA_(98-4); (**e**) G-B_GA_(98-9.8).

TEM images with smaller magnification at 10,000× are shown in Figure 8. The micrographs shown in Figure 8a–c have dark lines or zones corresponding to thick portion of the film due to cutting. Figure 8d shows a white area due to breaking of the film during cutting. However, the micrographs of G-B_GA_(98-4) and G-B_GA_(98-9.8) reveal dark spots different from those that represent larger domains of PS (circled in Figure 8d,e). The size of these PS domains are in the range of 0.5 to 2 μm. This explains the high haze (above 60%) and low clarity (below 20%), since light is diffracted and scattered. In contrast, there are no large dark regions visible in the micrographs of the samples G-B_GMA_(52-4) and G-B_GA_(55-4.7), correlating well with the lower haze (below 25%) and higher clarity (above 90%). G-B_GMA_(104-4.1) has higher haze and lower clarity compared to G-B_GMA_(52-4) and G-B_GA_(55-4.7). However, no large PS domains are detected in Figure 8b.

Based on the extent of the PS domain size in Figure 7b, some domains are larger and the continuity of the morphology is less evident compared to the transparent samples (Figure 7a,c). Therefore, it can be concluded that an increase of the molecular weight of the backbone affects the transparency of the blend by increasing the PS domain size.

## 4. Conclusions

The synthesis of PBA-g-PS copolymer blends is achieved by a three-step approach: (i) free-radical copolymerization of BA with GA or GMA to form PBA-co-GMA or PBA-co-GA copolymers; (ii) polymer analogous reaction with acrylic acid; and (iii) copolymerization of acryloyl modified backbones with styrene. It can be demonstrated that the type of comonomer used in the backbone synthesis (acrylate or methacrylate) influences the cross-linking reaction occurring during the grafting copolymerization. The acrylate-based comonomer reduces inhomogeneity in the density of the reactive groups, and therefore, the probability of cross-linking. This effect is particularly visible on backbones with molecular weight below 50 kg/mol and number of reactive groups of about 4 per chain.

Due to the high reactivity of acryloyl groups, their number has to be limited below 10 reactive sites per chain. A higher value leads to severe cross-linking affecting the film-casting process and optical properties of the blends.

SEC deconvolution method reveals differences in grafting efficiency between blends prepared with GA- or GMA-based backbones. The grafting efficiency decreases with the GMA-based backbone in dependence of reaction time; in the case of the GA-based backbones, the opposite tendency is observed. Increasing the molecular weight of the backbone from 50 to 100 kg/mol reduces the grafting efficiency and the transparency of the blends. Due to their morphology consisting of domains with sizes below 50 nm for all the graft copolymers, transparent products are obtained with haze as low as 14% and clarity as high as 99%. However, compared to PB-g-PS blends previously reported, the mechanical and optical properties of the novel PBA-g-PS/PS are still lower. This is due to the lower domain size of the PS phases (around 20 nm) in PB-g-PS/PS compared to PBA-g-PS/PS. Therefore, the main aim is to reduce the PS domain size to the critical value while keeping a homogeneous morphology.

Another significant difference lies in the grafting efficiency—while the novel PBA-g-PS/PS blends show grafting efficiencies up to about 35%, Portl reported grafting efficiencies for PB-g-PS/PS of about 60% [26].

Therefore, further increase in flexibility of PBA-g-PS/PS blends requires higher grafting efficiencies. At the same time, the cross-linking reaction needs to be suppressed efficiently. Varying the amount and the type of the reactive site in the backbone chain might be a way to enhance the mechanical and optical properties of these novel PBA-g-PS/PS copolymer blends further.

## Figures and Tables

**Table 1:** Structural variations for the synthesis of PBA-co-GA or PBA-co-GMA.

Backbone	Comonomer	TDT	BPO	M_n_	Đ	Wt % Comonomer	N_coM_
	-	[wt.% Rel. Monomer]		[kg/mol]	-	[wt.%]	-
B_GMA_(26-1.9)	GMA	0.75	1	26	2.8	1.92	3.5
B_GMA_(35-9)	GMA	0.75	1	35	2.9	9.00	22.2
B_GMA_(52-1.1)	GMA	0.25	1	52	3.0	1.10	4.0
B_GMA_(50-4.7)	GMA	0.25	1	50	2.6	4.73	16.6
B_GMA_(104-0.6)	GMA	0.20	0.1	104	2.2	0.56	4.1
B_GA_(55-1.1)	GA	0.25	1	55	2.6	1.10	4.7
B_GA_(98-0.5)	GA	0.20	0.1	98	1.9	0.52	4.0
B_GA_(98-1.3)	GA	0.20	0.1	98	2.0	1.28	9.8

**Table 2:** Number of co-monomers (GA or GMA, N_coM_) and number of acryloyl groups per chain in the backbones.

Backbone	Comonomer	N_coM_	N_Acr_
	-	-	-
B_GMA_(26-3.5)	GMA	3.5	3.5
B_GMA_(35-22)	GMA	22.2	22.0
B_GMA_(52-4)	GMA	4.0	4.0
B_GMA_(50-16.7)	GMA	16.6	16.7
B_GMA_(104-4.1)	GMA	4.1	4.1
B_GA_(55-4.7)	GA	4.7	4.7
B_GA_(98-4)	GA	4.0	4.0
B_GA_(98-9.8)	GA	9.8	9.8

**Table 3:** Non-soluble fraction and molecular weight distribution of PBA-g-PS/PS.

Blend	Backbone			Graft Copolymer			
	M_n_	N_Acr_	N_m_	[NS] %	M_n_	D	PS Content
	[kg/mol]	-	-	[wt.%]	[kg/mol]	-	[wt.%]
G-B_GMA_(26-3.5)	26	3.5	58	50	47	4.2	54
G-B_GMA_(35-22)	35	22.0	12	93	-	-	-
G-B_GMA_(52-4)	52	4.0	101	17	48	5.0	59.5
G-B_GMA_(50-16.7)	50	16.7	24	73	-	-	-
G-B_GMA_(104-4.1)	104	4.1	198	0	52	5.2	62.1

**Table 4:** Comparison of non-soluble fraction and molecular weight of copolymer blends based on GA and GMA backbones.

Blend	Backbone			Graft Copolymer		
	Mn	N_Acr_	N_m_	[NS] %	M_n_	PS Content
	[kg/mol]	-	-	[wt.%]	[kg/mol]	[wt.%]
G-B_GMA_(52-4)	52	4.0	101	17	48	59.5
G-B_GA_(55-4.7)	55	4.7	91	0	54	64.2
G-B_GMA_(104-4.1)	104	4.1	198	0	52	62.1
G-B_GA_(98-4)	98	4.0	192	0	49	65.3

**Table 5:** Mechanical and optical properties of the films.

Blend	Backbone				Graft Copolymer (Solvent-Cast Film)				
	Type of Comonomer	Mn	N_Acr_	N_m_	Haze	Clarity	E Modulus	Tensile Strength	Elongation at Break
		[kg/mol]	-	-	[%]	[%]	[MPa]	[MPa]	[%]
G-B_GMA_(52-4)	GMA	52	4.0	100.8	23.0 ± 3.9	91.5 ± 3.8	1010 ± 58	16.5 ± 1.2	3.4 ± 0.9
G-B_GMA_(104-4.1)	GMA	104	4.1	198.1	42.1 ± 0.6	38.9 ± 1.1	660 ± 153	19.7 ± 1.7	3.6 ± 0.3
G-B_GA_(55-4.7)	GA	55	4.7	90.9	13.6 ± 1.8	99.3 ± 0.1	1172 ± 147	21.9 ± 4.9	3.0 ± 0.6
G-B_GA_(98-4)	GA	98	4.0	192.2	69.1 ± 2.4	16.5 ± 2.1	Not measurable (too brittle)		
G-B_GA_(98-9.8)	GA	98	9.8	78.1	59.9 ± 3.8	7.9 ± 0.4	1034 ± 189	16.1 ± 3.0	1.3 ± 0.4

## References

[1] Adhikari R., Michler G.H., Lebek W., Goerlitz S., Weidisch R., Knoll K. (2002). Morphology and micromechanical deformation behavior of SB-block copolymers. II. Influence of molecular architecture of asymmetric star block copolymers. J. Appl. Polym. Sci..

[2] Chen C.C., White J.L. (1993). Compatibilizing agents in polymer blends: Interfacial tension, phase morphology, and mechanical properties. Polym. Eng. Sci..

[3] Knoll K., Nießner N. (1998). Styrolux^+^ and styroflex^+^ –from transparent high impact polystyrene to new thermoplastic elastomers: Syntheses, applications and blends with other styrene based polymers. Macromol. Symp..

[4] Kennedy J., Higginbotham C. (2011). Synthesis and Characterisation of Styrene Butadiene Styrene Based Grafted Copolymers for Use in Potential Biomedical Applications. Biomedical Engineering, Trends in Materials Science.

[5] Yang J.M., Tsai S.C. (2010). Biocompatibility of epoxidized styrene–butadiene–styrene block copolymer membrane. Mater. Sci. Eng.: C.

[6] Yang J.M., Huang H.T. (2012). Evaluation of tri-steps modified styrene-butadiene-styrene block copolymer membrane for wound dressing. Mater. Sci. Eng.: C.

[7] McKee G.E., Kistenmacher A., Goerrissen H., Breulmann M. (2003). Synthesis, Properties and Applications of Acrylonitrile–Styrene–Acrylate Polymers. Modern Styrenic Polymers: Polystyrenes and Styrenic Copolymers.

[8] Butté A., Storti G., Morbidelli M. (2003). Living Free Radical Polymerization of Styrene. Modern Styrenic Polymers: Polystyrenes and Styrenic Copolymers.

[9] Chrisochoou A., Dufour D. (2003). Styrenic Copolymers.

[10] Niessner N., Gausepohl H. (2003). Polystyrenes and Styrene Copolymers—An Overview. Modern Styrenic Polymers: Polystyrenes and Styrenic Copolymers.

[11] Listigovers N.A., Georges M.K., Odell P.G., Keoshkerian B. (1996). Narrow-Polydispersity Diblock and Triblock Copolymers of Alkyl Acrylates by a Living Stable Free Radical Polymerization. Macromolecules.

[12] Cassebras M., Pascual S., Polton A., Tardi M., Vairon J. (1999). Synthesis of di- and triblock copolymers of styrene and butyl acrylate by controlled atom transfer radical polymerization. Macromol. Rapid Commun..

[13] Hawker C.J., Bosman A.W., Harth E. (2001). New Polymer Synthesis by Nitroxide Mediated Living Radical Polymerizations. Chem. Rev..

[14] Matyjaszewski K., Shipp D.A., Qiu J., Gaynor G.S. (2000). Water-Borne Block and Statistical Copolymers Synthesized Using Atom Transfer Radical Polymerization. Macromolecules.

[15] Dawkins J.V., Davis K.A., Matyjaszewski K. (2002). Statistical, Gradient, Block and Graft Copolymers by Controlled/Living Radical Polymerizations.

[16] Arehart S.V., Matyjaszewski K. (1999). Atom Transfer Radical Copolymerization of Styrene and n-Butyl Acrylate. Macromolecules.

[17] Hong S.C., Pakula T., Matyjaszewski K. (2001). Preparation of Polyisobutene-graft-Poly(methyl methacrylate) and Polyisobutene-graft-Polystyrene with Different Compositions and Side Chain Architectures through Atom Transfer Radical Polymerization (ATRP). Macromol. Chem. Phys..

[18] Wang X., Luo Y., Li B., Zhu S. (2009). Ab Initio Batch Emulsion RAFT Polymerization of Styrene Mediated by Poly(acrylic acid-b-styrene) Trithiocarbonate. Macromolecules.

[19] Luo Y., Wang X., Zhu Y., Li B., Zhu S. (2010). Polystyrene-block-poly(n-butyl acrylate)-block-polystyrene Triblock Copolymer Thermoplastic Elastomer Synthesized via RAFT Emulsion Polymerization. Macromolecules.

[20] Weidisch R., Gido S.P., Uhrig D., Iatrou H., Mays J.W., Hadjichristidis N. (2001). Tetrafunctional Multigraft Copolymers as Novel Thermoplastic Elastomers. Macromolecules.

[21] Ito S., Goseki R., Ishizonea T., Hirao A. (2014). Synthesis of well-controlled graft polymers by living anionic polymerization towards exact graft polymers. Polym. Chem..

[22] Adhikari R., Lebek W., Godehardt R., Michler G.H.P.E.D.H., Cagiao M.E., Calleja F.J.B., Knoll K. (2006). Relationship between nanostructure and deformation behavior of microphase-separated styrene/butadiene systems. J. Appl. Polym. Sci..

[23] Dvořánek L., Vlček P. (1993). Anionic polymerization of acrylates. Polym. Bull..

[24] Vlcek P., Otoupalova J., Sikora A., Kriz J. (1995). Anionic Polymerization of Acrylates. 10. Synthesis and Characterization of Block Copolymers with Acrylate Blocks. Macromolecules.

[25] Dvoranek L., Vlcek P. (1994). Anionic Polymerization of Acrylates. 8. Kinetics of the Anionic Polymerization of Butyl Acrylate Initiated with the Complex Initiator Lithium Ester Enolate/Lithium tert-Butoxide. Macromolecules.

[26] Portl T. (2011). NanoHIPS als Schlagzähmodifizierter Thermoplast. Ph.D. Thesis.

[27] Huang N.-J., Sundberg D.C. (1994). A gel permeation chromatography method to determine grafting efficiency during graft copolymerization. Polymer.

[28] Ahmad N.M., Heatley F., Lovell P.A. (1998). Chain Transfer to Polymer in Free-Radical Solution Polymerization of n-Butyl Acrylate Studied by NMR Spectroscopy. Macromolecules.

[29] Siołek M., Matlengiewicz M. (2014). Reactivity Ratios of Butyl Acrylates in Radical Copolymerization with Methacrylates. Int. J. Polym. Anal. Charact..

[30] Catala J.M., Nonn A., Pujol J.M., Brossas J. (1986). Radical copolymerization of 2-hydroxyethylacrylate with alkylacrylate. Polymer Bulletin.

